# Extracellular vesicles from mast cells induce mesenchymal transition in airway epithelial cells

**DOI:** 10.1186/s12931-020-01346-8

**Published:** 2020-05-01

**Authors:** Yanan Yin, Ganesh Vilas Shelke, Cecilia Lässer, Hjalmar Brismar, Jan Lötvall

**Affiliations:** 1 Krefting Research Centre, Institute of Medicine at the Sahlgrenska Academy, University of Gothenburg, Gothenburg, Sweden; 2 Department of Biochemistry and Molecular Cell Biology, Shanghai Jiao Tong University, School of Medicine, 280 South Chongqing Road, Shanghai, 200025 China; 3 The Sahlgrenska Cancer Center, University of Gothenburg, Gothenburg, Sweden; 4 Department of Surgery, Institute of Clinical Sciences, University of Gothenburg and Sahlgrenska University Hospital, Gothenburg, Sweden; 5 Science for Life Laboratory, Dept. of Applied Physics, Royal Institute of Technology, PO Box 1031, 17121 Solna, Sweden

**Keywords:** Mast cells, Epithelial cells, Extracellular vesicles, Exosomes, Phosphorylated proteins, Protein microarray, Epithelial-mesenchymal transition

## Abstract

**Background:**

In the airways, mast cells are present in close vicinity to epithelial cells, and they can interact with each other via multiple factors, including extracellular vesicles (EVs). Mast cell-derived EVs have a large repertoire of cargos, including proteins and RNA, as well as surface DNA. In this study, we hypothesized that these EVs can induce epithelial to mesenchymal transition (EMT) in airway epithelial cells.

**Methods:**

In this in-vitro study we systematically determined the effects of mast cell-derived EVs on epithelial A549 cells. We determined the changes that are induced by EVs on A549 cells at both the RNA and protein levels. Moreover, we also analyzed the rapid changes in phosphorylation events in EV-recipient A549 cells using a phosphorylated protein microarray. Some of the phosphorylation-associated events associated with EMT were validated using immunoblotting.

**Results:**

Morphological and transcript analysis of epithelial A549 cells indicated that an EMT-like phenotype was induced by the EVs. Transcript analysis indicated the upregulation of genes involved in EMT, including *TWIST1*, *MMP9*, *TGFB1*, and *BMP-7*. This was accompanied by downregulation of proteins such as E-cadherin and upregulation of Slug-Snail and matrix metalloproteinases. Additionally, our phosphorylated-protein microarray analysis revealed proteins associated with the EMT cascade that were upregulated after EV treatment. We also found that transforming growth factor beta-1, a well-known EMT inducer, is associated with EVs and mediates the EMT cascade induced in the A549 cells.

**Conclusion:**

Mast cell-derived EVs mediate the induction of EMT in epithelial cells, and our evidence suggests that this is triggered through the induction of protein phosphorylation cascades.

## Background

Cells that are present in close proximity to each other, including immune cells recruited to epithelial tissues, are in constant communication with each other, both in disease or homeostatic condition [1–4]. This communication can be induced by direct cell-to-cell contact, secreted free proteins, or shuttling of bioactive molecules via extracellular vesicles (EVs). EV-based cellular cross talk is an evolutionarily conserved form of communication, and EVs can induce signaling through their cargoes of protein, DNA, and RNA [5–9]. With their varied cargo, EVs have the potential to activate multiple signaling events in recipient cells. However, how these different signaling events integrate together to drive a cellular phenotype is not well studied.

EVs have been reported to induce reprogramming of cells via epithelial-mesenchymal transition (EMT) [10–14]. During EMT, cells undergo programmed changes in cellular mRNA and protein content [15–17]. This process is highly coordinated and reversible and is associated with tissue damage and with numerous pathological conditions [18–21]. Extracellular cues recruit immune cells into the micro-niche of the lungs during chronic inflammatory diseases, including asthma, allergic reactions, and chronic obstructive pulmonary disorder [22–27]. During this process, lung epithelial cells are constantly exposed to inflammatory insults from recruited and resident immune cells, including mast cells [22, 28]. This damage induces structural re-arrangements of the epithelial lining along with tissue remodeling, increased metalloproteinase activity, the creation of fibrotic lesions, and altered cytokine levels [29–31]. Mast cells are known to interact physically with many cell types and thus to influence the inflammatory phenotype [29–31]. Some of the described inflammatory epithelial phenotypes are indeed features of the EMT process [32]. In this study we determined the potential of mast cell-derived EVs to regulate EMT, and we identified the potential signaling events in recipient epithelial cells.

In a previous study we showed that the mast cell-derived EV-associated membrane protein c-Kit is transferred to epithelial cells, resulting in downstream phosphorylation of AKT and GSK3-β and thus enhancing proliferation [33]. Our earlier studies also identified transforming growth factor (TGFβ-1) on the surface of mast cell-derived EVs that might play a role in EMT [34]. Here, we hypothesized that mast cell-derived EVs have the capacity to induce an EMT-like phenotype and that they activate multiple signaling events in lung epithelial cells. To this end, we used HMC1 cell derived EVs, as these cells are constitutively active and does not need growth-factor because of constitutive activity of the receptor tyrosine kinase Kit [35]. Our transcript, protein, and phosphoprotein analysis of epithelial cells showed activation of EMT pathways. This study highlights the potential signaling cascades activated by mast cell-derived EVs in regulating the EMT phenotype in epithelial cells.

## Methods

### Cell culture

The alveolar epithelial cell line A549 was obtained from ATCC, USA, and the cells were cultured in DMEM/F12 (Sigma Aldrich, St. Louis, MO, USA) supplemented with 10% fetal bovine serum (FBS; Sigma Aldrich). The culture media was changed to EV-depleted FBS-containing medium 24 h prior to the experiments. For all experiments the cells were seeded at a density of 15,000 cells/cm^2^.

Human mast cells, HMC-1 (J. Butterfield, Mayo Clinic, Rochester, MN, USA), were cultured in Iscove’s modified Dulbecco’s medium (HyClone Laboratories, Logan, UT, USA) supplemented with 10% EV-depleted FBS, 1.2 mM α-thioglycerol (Sigma Aldrich), and 2 mM L-glutamine (HyClone Laboratories). These cultures were supplemented with 100 units/ml penicillin and 100 μg/ml streptomycin (HyClone Laboratories) and cultured at 37 °C in a 5% CO_2_ humidified conditions. The FBS used for HMC-1 cultures was ultracentrifuged for 18 h at 120,000×*g* (Type 45 Ti rotor, Beckman Coulter) to remove the serum EVs, as reported earlier [36].

### Isolation of EVs

Conditioned medium from HMC-1 cells was obtained after 3–4 days of culture, and cells were removed by centrifugation at 300×*g* for 10 min. The cell-free supernatant was further centrifuged at 16,500×*g* for 20 min to remove microvesicles and apoptotic bodies. Finally, this supernatant was centrifuged at 120,000×*g* for 3 h (Type 45 Ti rotor, Beckman Coulter), and the pelleted EVs were washed once with PBS. The final EV pellet was suspended in PBS and stored at − 80 °C for further experiments. The protein concentration of the EVs was measured using the BCA protein assay kit (Pierce, Thermo Fisher Scientific, Waltham, MA, USA).

### EV labeling and cellular uptake

EVs obtained from HMC-1 cells were labeled with the PKH67 Green Fluorescent Cell Linker Kit (Sigma Aldrich) as per the manufacturer’s protocol. The labeled EVs were loaded onto the bottom of an iodixanol density gradient (0, 20, 30, and 50% iodixanol) and centrifuged at 28,000 rpm for 2 h in a swinging bucket rotor (SW40Ti, Beckman Coulter). The EVs floating over the interphase (20–30%) were collected and washed in PBS followed by centrifugation at 120,000×*g* for 3 h (Type 45 Ti rotor, Beckman Coulter). A549 cells were grown on coverslips at 15,000 cells/cm^2^ for 24 h. The labeled EVs were incubated with the A549 cells grown on the coverslip for 2 h or for 16 h. The cell membranes and nuclei were stained with the Image-IT LIVE kit (Invitrogen, Thermo Fisher Scientific) using Alexa Fluor-594 wheat germ agglutinin and Hoechst 33342, respectively, according to the manufacturer’s protocol. The cells were fixed in a paraformaldehyde (3.5%) solution for 10 min and washed before the cover slip containing the cells was mounted on a slide and imaged under a structural illumination microscope (Zeiss Elyra 3D SIM, Germany).

### Gelatin zymography

A549 cells were exposed to mast cell-derived EVs, and conditioned medium was collected at 24 h and at 48 h. The conditioned media was separated on gelatin-contacting zymogram gels (BioRad Laboratories, Hercules, CA, USA) with 5× non-reducing loading buffer (Sigma Aldrich). Renaturation of matrix metalloproteinases in the gel was performed at room temperature in 2.5% Triton X-100 (Sigma Aldrich) for 1 h followed by overnight incubation at 37 °C in development solution (50 mM Tris (pH 7.4), 5 mM CaCl_2_, 200 mM NaCl). Gels were then stained with Coomassie brilliant blue and destained (30% methanol and 10% acetic acid) until the white bands that reflect gelatinase activity appeared. Finally, 2% acetic acid was added to stop the destaining process. The degree of gelatinase activity was measured by quantifying the band intensity using ImageJ software.

### Reversed cell migration assay

The Boyden chamber migration assay (Neuroprobe, Gaithersburg, MD, USA) was used to determine the migratory potential of A549 cells upon EV stimulation as described earlier [34].

### Immunofluorescence microscopy

A549 cells were incubated with EVs for 24 h before being fixed with 3.7% paraformaldehyde at room temperature for 10 min, permeabilized for 5 min with 0.2% Triton X-100, washed, and blocked for 1 h in 3% BSA. Finally, incubation with primary antibody for N-cadherin (NCAD) was performed for 1 h at room temperature, and the sample was washed again before it was stained with AF488-labeled secondary antibody. After three washes with PBS, the cells were further stained with DAPI (Sigma Aldrich) and cover slips were mounted using ProLong Gold anti-fade mounting reagent (Invitrogen, Carlsbad, CA, USA) and observed under a fluorescence light microscope (Axio Observer, Zeiss, Oberkochen, Germany).

### Secretion of TGFβ-1 measured by ELISA

The amount of TGFβ-1 secreted by A549 cells was measured by TGFβ-1 ELISA Ready-SET-Go kit (eBioscience Affymetrix) according to the manufacturer’s instructions.

### Quantitative real time PCR

RNA from epithelial A549 cells was isolated using the column-based miRCURY™ RNA isolation kit for cell and plant (Exiqon, Vedbaek, Denmark) and treated with TURBO DNase (Ambion, Life Technologies) to remove contaminating DNA. The concentration and purity of the RNA was quantified using a NanoDrop system (Thermo Scientific). The iScript cDNA Synthesis Kit (BioRad) was used to generate cDNA from 200 ng RNA. SsoAdvanced Universal SYBR Green Supermix was used to perform quantitative real time PCR in a BioRad CFX96 system for data collection and analysis. Briefly, cDNA was denatured at 95 °C for 30 s followed by 40 cycles of 95 °C for 15 s and 60 °C for 30 s. KiCqStart® primers (Sigma) were obtained for the following genes: *TGFB1*, *TWIST1*, *SMAD2*, *MMP2*, *WNT5A*, *FOXC2*, *BMP7*, and *VIM*. The endogenously expressed *EF1* gene was used for transcript normalization. Relative fold change in gene expression was calculated by the 2_T_^−ΔΔC^ method.

### Western blotting

A549 cells were seeded at 0.2 × 10^6^ cells/well in 6-well plates and incubated for 24 h. Cells were then treated with 30 μg/ml EVs or 10 ng/ml TGFβ-1 at different time points. The A549 cell pellet was washed in PBS, and the cells were lysed in 1× RIPA buffer (Cell Signaling Technology ST, Danvers, MA, USA) with 1× Halt protease and phosphatase inhibitor cocktail (Halt, Thermo Fisher Scientific). Protein lysates were subjected to SDS-PAGE and transferred onto nitrocellulose membranes. Nonspecific binding sites were blocked with Tris-buffered saline with 0.05% Tween-20 and 5% non-fat milk or 5% bovine serum albumin (BSA) for 1 h at room temperature. Primary antibodies in their respective blocking buffer were incubated at 4 °C overnight. Horseradish peroxidase-conjugated secondary antibodies (1:10,000 dilution, NA931V, NA9340, NA9310V, GE Healthcare) were diluted in blocking buffer and incubated with the membrane for 1 h at room temperature. Chemiluminescence signal for proteins was detected with SuperSignal West Femto Maximum Sensitivity Substrate (Thermo Fisher Scientific) according to the manufacturer’s protocol. The following antibodies were used: NCAD (3B9; 1:1000 dilution, #33–3900 Invitrogen, Thermo Fisher Scientific), E-cadherin (ECAD) (1:1000 dilution, #610182, BD Biosciences), Slug-Snail (1:1000 dilution, #ab180714, Abcam), and β-actin (1:3000 dilution, #sc47778, Santa Cruz Biotechnology).

### Phospho-proteomics microarray

#### Sample preparation

Sample processing and analysis was performed as per the guidelines provided by Sciomics (Heidelberg, Germany). Epithelial A549 cells were seeded at a density to achieve ~ 70% confluency in 24 h. Approximately 2 million cells were either treated with EVs (30 μg/ml) or left untreated. Each set was a biological duplicate (i.e. two untreated and two EV treated). After 60 min, the cells were rinsed with PBS and pelleted. Pelleted samples were snap-chilled in dry ice and stored at − 80 °C until use and sent to Sciomics for further analysis. Extraction of proteins was performed with proprietary scioExtract buffer (Sciomics), and the protein concentration was measured using the BCA assay. Samples were labeled with scioDye for 1 h adjusting the protein concentration, and the reaction was then stopped by the addition of hydroxylamine. Excess dye was removed 30 min later and the buffer was exchanged to PBS. All labeled protein samples were used immediately.

#### Antibody microarrays

The samples were analyzed using a scioDiscover antibody microarray (Sciomics) targeting 1033 different proteins with 1516 antibodies. Each antibody was represented on the array in four replicates. The arrays were blocked with scioBlock (Sciomics) on a Hybstation 4800 (Tecan, Austria). The antibodies were first added to the microarray and then the samples and scioPhosphomix were incubated. scioPhosphomix provides information on protein-specific phosphorylation levels of serine, threonine, and tyrosine residues. After incubation for 3 h, the slides were thoroughly washed with 1× PBS (with Tween-20 and Triton X-100), rinsed with 0.1× PBS, rinsed with water, and dried under nitrogen.

#### Data acquisition and analysis

Slide scanning was performed with a Powerscanner (Tecan, Austria) with identical instrument laser power and adjusted photomultiplier tube settings. Spot segmentation was performed with GenePix Pro 6.0 (Molecular Devices, Union City, CA, USA). Acquired raw data were analyzed using the linear models for microarray data (LIMMA) package of R-Bioconductor after uploading the median signal intensities. For normalization, a Cyclic Loess normalization was applied. For analysis of the samples, a one-factorial linear model was applied with LIMMA resulting in a two-sided t-test or F-test based on moderated statistics. All presented *p*-values were adjusted for multiple testing by controlling for the false discovery rate according to Benjamini and Hochberg. Proteins were defined as differentially expressed with an IlogFCI > 0.5 and an adjusted p-value < 0.05. Differences in protein abundance or phosphorylation level between different samples or sample groups are presented as log-fold changes (logFC) calculated for base 2. In a study comparing samples versus controls, a logFC = 1 means that the sample group had on average a 2^1^ = 2-fold higher signal than the control group. logFC = − 1 stands for 2^− 1^ = 1/2 of the signal in the sample compared to the control group.

## Results

### Morphological changes are induced in epithelial cells upon uptake of mast cell-derived EVs

**Fig. 1:**
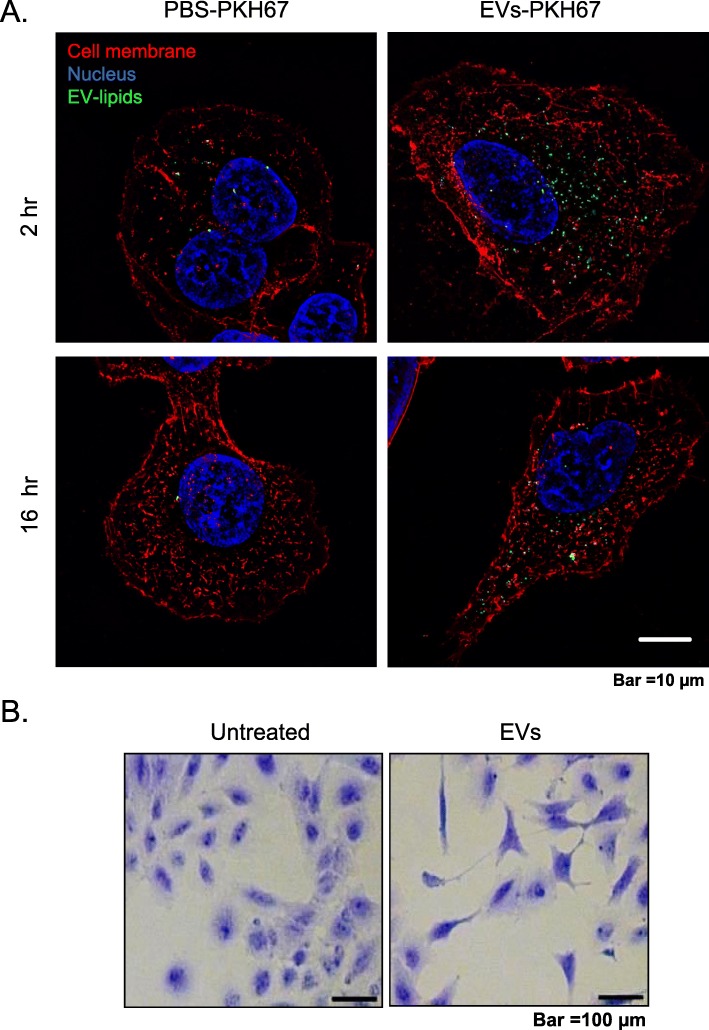
Epithelial cells take up mast cell-derived EVs and undergo morphological changes. **a** High-resolution fluorescence imaging of A549 cells at 2 h and 16 h that have taken up HMC-1-derived EVs (10 μg/ml). Labels: EV lipids (green, PKH67), cell membrane (red, Alexa Fluor 594), and nucleus (blue, Hoechst 33342). **b** Morphological assessment of Coomassie brilliant blue-stained A549 cells after 24 h of EV treatment (30 μg/ml). Representative images are shown from two independent experiments

Mast cells are known to reside in the proximity of airway epithelial cells and can therefore readily interact with epithelial cells. To study EV-mediated cross talk between these cell types, we used an in vitro system in which A549 lung epithelial cells were exposed to mast cell-derived EVs (HMC-1) labeled with a lipid dye (PKH67). Super resolution microcopy showed that the fluorescent signal from the EVs was taken up and retained by the A549 epithelial cells at both 2 h and 16 h, indicating that epithelial cells can integrate mast cell-derived EVs (Fig. 1a). Furthermore, prolonged co-culturing with mast cell-derived EVs induced morphological changes in the A549 cells, including elongated protrusions (Fig. 1b).

### Mast cell-derived EVs upregulate EMT-related transcripts in epithelial cells

**Fig. 2:**
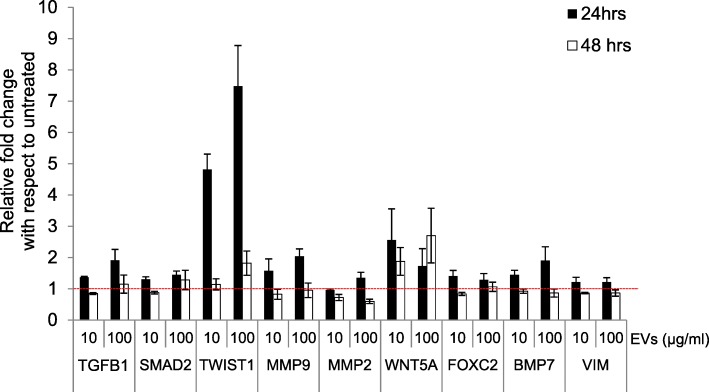
EVs induce the expression of EMT genes in lung epithelial cells. Transcript levels of genes involved in EMT were evaluated in A549 epithelial cells at 24 h and 48 h with different dosages of EVs. The dotted red line indicates the baseline expression of transcripts to which the fold changes are plotted. The transcript levels are normalized with respect to the housekeeping gene *EF-1*. *N* = 3 or 4, and the results are presented as the average ± SEM

The observed morphological changes in A549 cells led us to hypothesize that EVs can induce EMT. To test this, the expression of several genes involved in EMT such as *TGFB1*, *SMAD2*, *TWIST1*, *MMP9*, *MMP2*, *WNT5A*, *FOXC2*, *BMP7*, and *VIM* were evaluated in A549 cells. The levels of most of the transcripts (especially *TGFB1*, *TWIST1 MMP9*, and *BMP7*) were upregulated by EV exposure in a dose and time-dependent manner (Fig. 2). These transcripts increased initially at 24 h after exposure to the EVs and began to decrease by 48 h.

### A mesenchymal-like phenotype is induced in epithelial cells by EVs

**Fig. 3:**
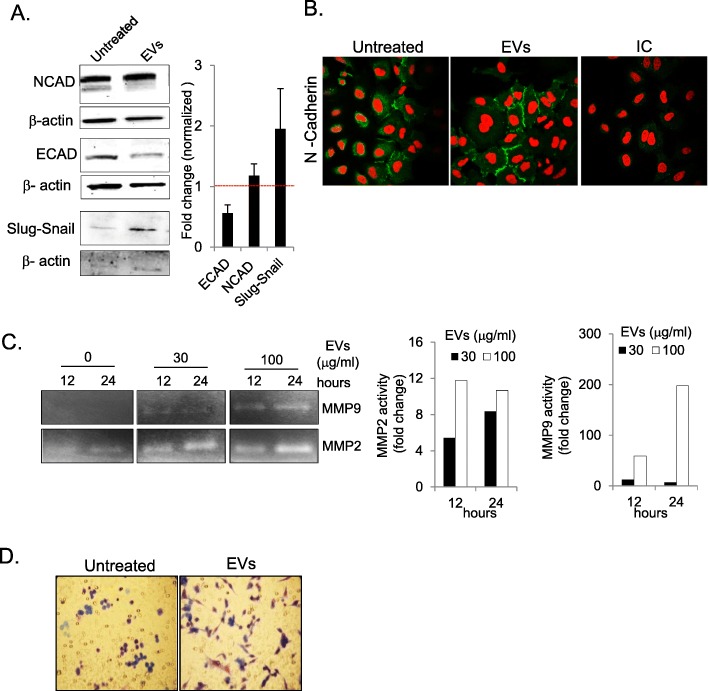
EVs induce an EMT-like phenotype in lung epithelial cells. **a** Immunoblotting analysis of NCAD, ECAD, and Slug-Snail protein in A549 cells after 24-h incubation with EVs (30 μg/ml). Representative images are shown from three independent experiments. **b** Immunofluorescence imaging of A549 cells to determine the cellular distribution of NCAD (green) and DAPI (red). Images were acquired at 20× magnification after 48 h of EV treatment (30 μg/ml). **c** Gelatin zymography analysis of culture supernatant of EV-treated A549 cells to determine the matrix metalloproteinase activity of MMP-9 and MMP-2. **d** Boyden chamber reverse trans-well migration assay of epithelial cells towards EVs (30 μg/ml). Representative images are shown from three independent experiments

Another feature that has been studied in EMT progression is the downregulation of the epithelial protein ECAD and the upregulation of the mesenchymal protein NCAD [10]. At the total protein level we observed a decrease in ECAD, but NCAD levels seemed overall unchanged (Fig. 3a). To determine whether NCAD in the A549 cells was associated with the cell membrane after EV exposure, we performed immunofluorescence microscopy and observed increased expression at the cellular junctions (Fig. 3b). Moreover, the expression of Slug-Snail, a protein reported to be critical for EMT, was also increased in the EV-treated samples (Fig. 3a). Additionally, the EMT phenotype is associated with an increase in matrix-degrading proteases [37, 38], and in the current study we observed increased MMP-2 and MMP-9 activity in the extracellular milieu of the A549 cells after EV exposure, as detected by gelatin-zymography (Fig. 3c). This was also accompanied by enhanced invasion of A549 cells towards EVs in a reverse-Boyden chamber trans-migration assay (Fig. 3d). Taken together, these findings suggest the activation of EMT.

### EVs induce the phosphorylation of multiple proteins in epithelial cells

**Fig. 4:**
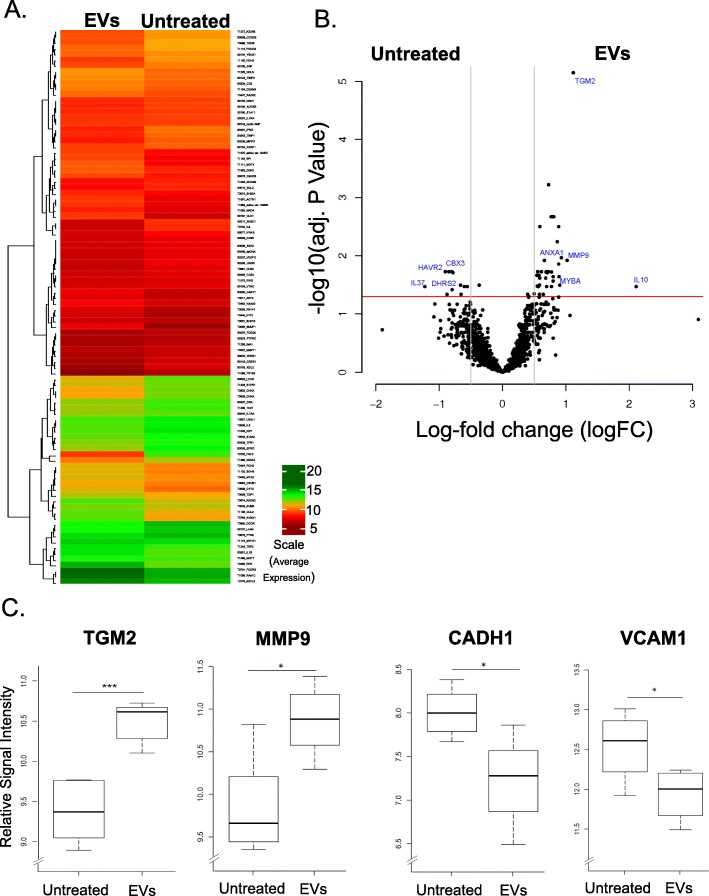
Analysis of phosphorylated proteins in EV-treated lung epithelial cell. **a** Heat map of commonly phosphorylated protein found in A549 cells treated with 30 μg/ml of EVs vs. untreated. **b** Several proteins exhibited distinct phosphorylation variations in EV-treated and untreated cells. The volcano plot visualizes the *p*-values (adjusted for multiple testing) and corresponding log-fold changes. An adjusted p-value of 0.05 is indicated as horizontal line. Proteins with a positive log-fold change were more phosphorylated in EV-treated cells, and proteins with a negative log-fold change were more phosphorylated in untreated cells. **c** Individual array values of differentially phosphorylated proteins (TGM2, MMP-9, CADH1, and VACM) in A549 cells after EV treatment. Each sample was measured by four replicate spots per array

The proteins that we evaluated for EMT were studied at 24 and 48 h (Fig. 2 and Fig. 3); however, the effects seen at the transcript and protein level were due to early signaling events activated by the EVs. Therefore, we sought to determine the multiple signaling pathways that are activated by multi-cargo EVs in epithelial cells after 30 min. To assess the signaling, we monitored serine/threonine protein phosphorylation, which is the most common post-translational modification. We used an antibody-based microarray platform that cataloged 1033 different proteins with 1516 antibodies. A total of 96 antibodies recorded a differentially phosphorylated proteins between EV-treated and untreated cells. Heat map analysis of all identified phosphorylated proteins in the A549 cells suggested that none of the changes were due to sample treatment and processing (Fig. 4a). Of these phosphorylated proteins, 47 (~ 50%) were differentially phosphorylated after EV treatment compared to untreated A549 cells. A few of these phosphorylated proteins are listed in Table 1. In total, 35 phosphorylated proteins had a fold change > 1.4 and were considered to be up-phosphorylated, and the remaining 12 with a fold change < -1.4 were considered to be down-phosphorylated. The changes were statistically different with adjusted *p*-values < 0.05 (duplicates in two independent experiments with signal from four antibody spots, Fig. 4b). To visualize the data from the table, we plotted the relative signal intensity profiles of two up-phosphorylated proteins (TGM2 and MMP-2) and two down-phosphorylated proteins (CADH1 and VCAM-1) (Fig. 4c) [39–42]. These proteins regulate the EMT process, and thus our results support their possible role in modulating EMT.

**Table 1:**
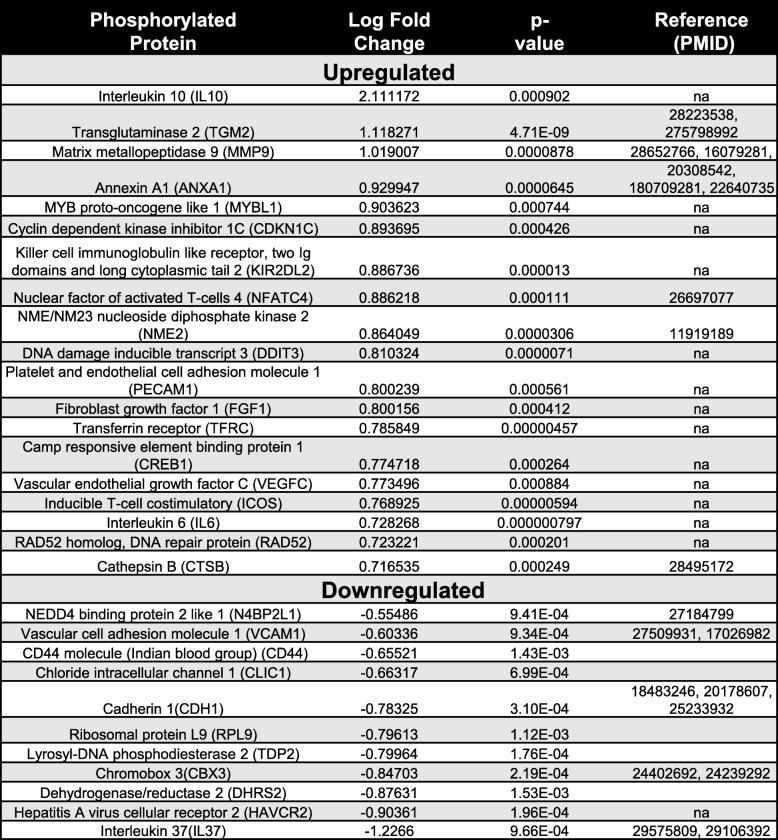
Differentially regulated phosphoproteins in A549 cells with or without EV exposure and their roles in epithelial to mesenchymal transition reported in the literature. Proteins with a positive logFC value were more phosphorylated in EVs, and proteins with a negative value were more phosphorylated in untreated cells. In addition, the p-values adjusted for multiple testing are listed

The activation of A549 cells by EVs led us to ask whether the relative abundance of various proteins and not just their phosphorylation might be regulated by the signaling networks within the cells. *In-silico* protein classification revealed a number of proteins that are known to regulate the PI3K-Akt signaling pathway (e.g. AKT3, BCL2, CCND2, CREB1, NFKB1, EGF, and IL6) and the HIF-1 pathway (e.g. CUL2, IFNG, IGF1, TFRC, and TIMP1) as well as proteins with direct cytokine-cytokine receptor interactions (e.g. TNFRSF13B, VEGFC, EGF, IFNG, IL1B, IL7R, IL8, and IL2RA) as described in supplementary Table 1. Some of the upregulated proteins (e.g. AKT3, CCND2, PARP1, IL6, NFKB1, and IL2RA) are known to be involved in other pathways, including NFĸB, JAK, and STAT signaling. Sets of proteins regulating focal adhesion and tight junctions were also identified in these experiments (e.g. CLDN1, OCLN, and ACTN1).

The first opportunity for an EV to influence a recipient cell is at the moment of surface-to-surface contact. To determine which of the proteins might be acting at the cell surface, we classified the phosphorylated proteins based on their respective cellular components (Supplementary Table 2). We identified the association of several proteins with membrane-bound organelles, vesicles, and organelle lumen-associated structures, indicating that the EV-associated cargo engages with the epithelial cell membrane and induces the activation of the membrane compartment or a membrane- associated protein.

### EV-associated TGFβ-1 induces TGF signaling in epithelial cells

**Fig. 5:**
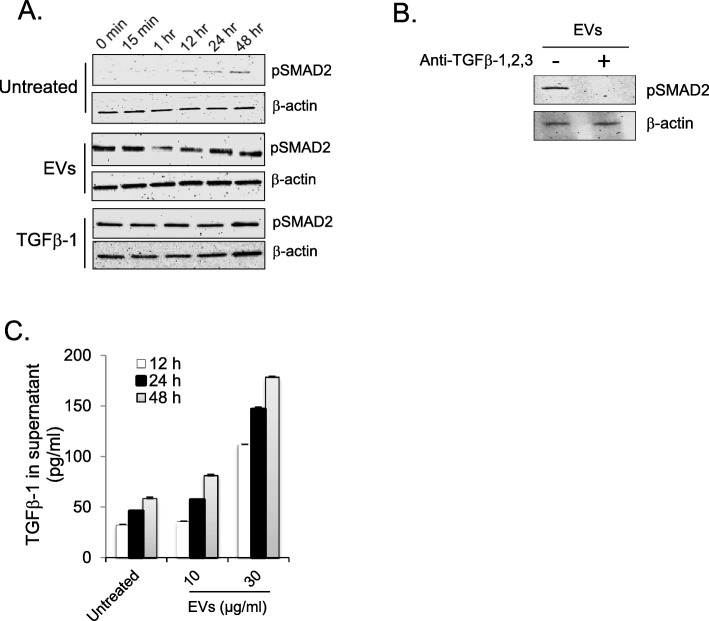
EVs induce TGF signaling in A549 epithelial cells. **a** Immunoblotting of phosphorylated SMAD2 expression over time in epithelial cells after incubation with 30 μg/ml of EVs and 10 ng/ml of TGFβ-1. Representative images are shown from three independent experiments. **b** Immunoblot analysis of phosphorylated SMAD2 in A549 cells exposed to EVs after pre-treatment with a blocking antibody against TGFβ-1. Representative images are shown from two independent experiments. **c** TGFβ-1 levels in the culture supernatants from epithelial cells that were exposed to EVs, as detected by ELISA

In our previous study [34] we showed the presence of TGFβ-1 on mast cell-derived EVs. Because TGFβ-1 itself can induce EMT in epithelial cells [23], we performed immunoblotting analysis of TGFβ-1-induced phosphorylation of SMAD2 in EV-treated A549 cells. In a time-based kinetics experiment, we observed a rapid increase in SMAD2 phosphorylation within the first several minutes, which decreased at 60 min, and then increased again at 24 h (middle panel, Fig. 5a). Untreated samples showed no phosphorylation of SMAD2 at 1 h (upper panel, Fig. 4a), but after 12 h and onwards a steady increase in phospho-SMAD2 was observed (upper panel, Fig. 4a). Additionally, we could see similar activation of phospho-SMAD2 in the bronchiolar epithelial cell line BEAC2B after EV treatment (Supplementary Figure 1). Constitutive activation of phospho-SMAD2 in epithelial cells was observed along with active TGFβ-1 (10 ng/ml) (lower panel, Fig. 5a). The activation of SMAD by phosphorylation mediated by EVs was reduced when EVs were pre-incubated with a blocking antibody against TGFβ-1 (Fig. 5b). We further observed an increase of *TGFB1* transcripts in EV-exposed A549 cells (Fig. 2), and it has previously been shown that TGFβ-1 induces its own production in an autocrine manner [43]. Further, we observed that the EVs increased the release of TGFβ-1 in a dose and time-dependent manner (Fig. 5c).

## Discussion

Epithelial cells in the lungs act as barrier and maintain homeostasis as they respond to extracellular factors from neighboring immune cells, including mast cells, and to foreign antigens [44–46]. In the current study, we observed that mast cell-derived EVs were able to induce migration of the airway epithelial cell line A549 in-vitro, and this was accompanied by multiple indicators of EMT. We also demonstrated the differential regulation of EMT-associated transcripts (*TWIST1*, *TGFB1*, *MMP9*, and *MMP2*) and protein markers (MMP, Slug-Snail, NCAD, and ECAD) in response to the mast-cell EVs. We also found that EVs mediated the early signaling response by inducing the phosphorylation of multiple proteins in epithelial A549 cells that have been suggested to be involved in regulating EMT. For example, TGFβ-1 present on EV surfaces acts as an early signal to induce the phosphorylation of SMAD2 in A549 cells. Taken together, the results of this study show that mast cell-derived EVs induce an EMT response in epithelial cells, and this phenotypic cell response appears to be due to the early phosphorylation of numerous proteins known to be involved in EMT.

Various immune cells, including mast cells, T-cells, B-cells, and dendritic cells, are known to produce EVs under various inflammatory conditions [7, 47–49]. Mast cells have been referred to as the “rheostat” of the local immune system, and they can release factors for host defense as well as for processes such as remodeling, wound-healing, angiogenesis, and cancer progression [50–52]. The number of mast cells is high in the vicinity of epithelial cells in the peripheral airways, and this proximity provides an opportunity for cell-to-cell cross talk between mast cells and epithelial cells [44]. In our in vitro studies, we found that mast cell-derived EVs were taken up by epithelial cells at different time points (Fig. 1a). Furthermore, the uptake of these EVs by epithelial cells induced a morphological change with elongated protrusions (Fig. 1b). The A549 cells showed detachment from neighboring cells after incubation with mast cell-derived EVs, and this was associated with upregulation of EMT-promoting markers (Fig. 2 and Fig. 3 a). An earlier study by Kasai et al. showed similar morphological changes in A549 cells and molecular changes in line with EMT induced by free TGFβ-1 [23]. However, in the current study, the induction of EMT by EVs was observed as early as 24 h after treatment, compared to 48 h in the previous study in which free TGFβ-1 was given. In our previous study, we showed that TGFβ-1 is present on the surface of EVs, resulting in potent activation of TGF signaling in mesenchymal stem cells [34]. Similarly, in the present study we observed the activation of SMAD2 in both alveolar A549 and bronchial BEAS-2B epithelial cells (Fig. 4 and Supplementary Figure 1).

EV membranes harbor multiple proteins, including growth factors that have bioactive functions. Interactions between TGFβ-1 and the EV surface are partially due to the glycoprotein-like heparin and heparan sulfate glycosaminoglycan present on the EV surface [53]. These glycoproteins are known to interact with multiple growth factors like TGFβ-1, FGF and VEGF [54–56]. It is likely that EV-associated TGFβ-1 activates A549 cells to initiate early EMT-inducing pathways; however, other molecular pathways might also be involved in activating EMT. Mast cell-derived EVs contain other secreted proteins like tryptase α/β-1, galectin-1, proteoglycan-2, and macrophage migration inhibitory factor that could potentially have additive effects [34]. Further, DNA present on the mast cell EV surfaces might also be involved in the biological activity of the released EVs [57]. A recent study by Kobayashi et al. showed that extracellular CpG DNA in combination with TGFβ-1 enhanced the EMT cascade in A549 cells at lower concentrations compared to TGFβ-1 alone. This suggests that EVs and multiple associated cargos, including TGFβ-1 and surface-exposed DNA, might be driving the epithelial phenotype because they can interact with cells simultaneously. However, the role of EV surface-associated DNA in the regulation of EMT as suggested in this study remains to be confirmed in further studies.

To evaluate whether the morphological changes induced in epithelial cells via EVs were really due to EMT, we analyzed the transcripts of a set of genes reported to be involved in the induction of EMT. We observed significant increases in the transcript levels of *TWIST1*, *TGFB1*, *MMP9*, and *BMP7*, all of which are known to be involved in EMT. In parallel, we observed an increase in proteins involved in EMT such as TGFβ-1, Slug-Snail, MMP-2, and MMP-9. Interestingly, the increase in these mRNA levels was clear at the 24 h time point, but diminished at 48 h, possibly because the EMT response induced by the EVs is only transient. However, we observed a continuous increase in the release of TGFβ-1 in A549 supernatant up until 48 h post-EV exposure, suggesting that some biological functions might be longer lasting than others. This suggests that TGFβ-1 protein production might be important for the initiation but not for the maintenance of EMT (with respect to *TGFB1* transcripts).

The EMT response is driven by upregulation of NCAD and the downregulation of ECAD. After the stimulation of epithelial cells with EVs, we observed a reduction in total protein for the epithelial marker ECAD, whereas levels of NCAD remained unchanged. It is possible that the cellular distribution of NCAD might have been altered, and thus we performed immunofluorescence microcopy to determine the cellular location of this molecule in epithelial cells after co-incubation with EVs. Indeed, we observed higher NCAD expression at the cellular junctions, indicating that EVs altered the cellular distribution of this molecule in the recipient cells. Previous studies have suggested that the presence of surface NCAD is associated with a higher migratory phenotype compared to higher levels of cytoplasmic NCAD [58, 59], and this is supported by our present findings. Thus, based on this observation, we suggest that NCAD present on the cell surface can be sufficient to induce an EMT-associated migratory response. This migratory capacity is coupled to enhanced secretion of bioactive matrix metalloproteinases because the reduction of matrix component might indirectly assist cellular movement [33].

With our current findings on EMT and previous observations that mast cell-derived EVs can induce A549 cell proliferation, we hypothesize that multiple cargos present on mast cell-derived EVs can activate multiple signaling cascades in recipient cells [33]. Indeed, mast cell-derived EVs induced the phosphorylation of at least 47 different cellular proteins in epithelial A549 cells. Importantly, a majority of these are reported to be involved in EMT, for example, upregulation of TGM2, MMP-9, and Annexine-A1 and downregulation of VACM1, CADH1, and Chrombox3 proteins.

Beyond phosphorylation of EMT-associated proteins, we also observed phosphorylation of proteins involved in associated pathways, such as the PI3K-AKT, HIF-1, NFĸB, and Jak-STAT signaling pathways. In our previous study, we described the transfer of constitutively active c-KIT membrane receptor from mast cell-derived EVs to epithelial cells, and this was associated with the activation of PI3K-AKT signaling. Indeed, PI3K-AKT signaling can be activated by multiple upstream factors, including multiple growth factor such as TGFβ-1, in addition to the c-KIT transfer [33]. PI3K-AKT signaling is central to many of the signaling pathways that induce EMT-like processes, so these results were not surprising. In this study we wanted to highlight the possibility of multiple signaling pathways activated by mast cell-derived EVs instead of focusing on a single pathway. A question that has remained unanswered is how the multiple and converging cellular responses that EVs can induce lead to a specific phenotype change such as EMT in recipient cells. Interestingly, we observed changes in the phosphorylation of proteins that regulate cell-to-cell contact, e.g. focal adhesion, cell adhesion, and tight junction proteins, and such changes might lead to rapid alterations of cell surface dynamics.

## Conclusion

In conclusion, this study provides evidence that mast cell-derived EVs could induce an EMT phenotype in lung epithelial A549 cells, and it highlights the importance of the cellular responses that are induced in the recipient cells. Our results suggest the ability of EVs and their associated cargo to initiate protein phosphorylation events in epithelial cells, and these events can potentially activate multiple cellular pathways that converge in an EMT or associated phenotype.

## Supplementary information

**Additional file 1: Figure S1.** Activation of SMAD in the bronchial epithelial cell line BEAS-2B after treatment with 30 μg/ml of EVs at 24 and 48 h. **Table S1.** Selected KEGG pathways related to proteins with differential abundance in epithelial A549 cells with or without EV treatment. **Table S2.** Selected cellular components related to proteins with differential abundance in epithelial A549 cells with or without EV treatment. The protein count indicates the number of proteins shown to engage with those cellular components.

## Data Availability

The data for the study are available from the corresponding authors on reasonable request.

## Abbreviations

EMT: Epithelial to mesenchymal transition
FBS: Fetal bovine serum
BCA: Bicinchoninic acid
SIM: Structured illumination microscope
DAPI: 4′, 6-diamidino-2-phenylindole
AF488: Alexa Fluor 488
PBS: Phosphate-buffered saline
BSA: Bovine serum albumin
ELISA: Enzyme-linked immunosorbent assay
ECAD: Epithelial cadherin
NCAD: Neural cadherin
RIPA: Radio immuno-precipitation assay
LIMMA: Linear models for microarray
LogFC: Log-fold changes

## References

[1] Hsieh FH, Sharma P, Gibbons A, Goggans T, Erzurum SC, Haque SJ (2005). Human airway epithelial cell determinants of survival and functional phenotype for primary human mast cells. Proc Natl Acad Sci U S A.

[2] Sanmugalingam D, Wardlaw AJ, Bradding P (2000). Adhesion of human lung mast cells to bronchial epithelium: evidence for a novel carbohydrate-mediated mechanism. J Leukoc Biol.

[3] Al-Ghadban S, Kaissi S, Homaidan FR, Naim HY, El-Sabban ME (2016). Cross-talk between intestinal epithelial cells and immune cells in inflammatory bowel disease. Sci Rep.

[4] Wells JM, Rossi O, Meijerink M, van Baarlen P (2011). Epithelial crosstalk at the microbiota-mucosal interface. Proc Natl Acad Sci U S A.

[5] Cossetti C, Iraci N, Mercer TR, Leonardi T, Alpi E, Drago D (2014). Extracellular vesicles from neural stem cells transfer IFN-gamma via Ifngr1 to activate Stat1 signaling in target cells. Mol Cell.

[6] Lee TH, Chennakrishnaiah S, Audemard E, Montermini L, Meehan B, Rak J (2014). Oncogenic ras-driven cancer cell vesiculation leads to emission of double-stranded DNA capable of interacting with target cells. Biochem Biophys Res Commun.

[7] Raposo G, Nijman HW, Stoorvogel W, Liejendekker R, Harding CV, Melief CJ (1996). B lymphocytes secrete antigen-presenting vesicles. J Exp Med.

[8] Ashley J, Cordy B, Lucia D, Fradkin LG, Budnik V, Thomson T (2018). Retrovirus-like gag protein Arc1 binds RNA and traffics across synaptic boutons. Cell.

[9] Valadi H, Ekstrom K, Bossios A, Sjostrand M, Lee JJ, Lotvall JO (2007). Exosome-mediated transfer of mRNAs and microRNAs is a novel mechanism of genetic exchange between cells. Nat Cell Biol.

[10] Greening DW, Gopal SK, Mathias RA, Liu L, Sheng J, Zhu HJ (2015). Emerging roles of exosomes during epithelial-mesenchymal transition and cancer progression. Semin Cell Dev Biol.

[11] Garnier D, Magnus N, Lee TH, Bentley V, Meehan B, Milsom C (2012). Cancer cells induced to express mesenchymal phenotype release exosome-like extracellular vesicles carrying tissue factor. J Biol Chem.

[12] Garnier D, Magnus N, Meehan B, Kislinger T, Rak J (2013). Qualitative changes in the proteome of extracellular vesicles accompanying cancer cell transition to mesenchymal state. Exp Cell Res.

[13] Rosic B, Sulovic V, Juznic N, Lazarevic B, Milacic D, Vidanovic M (1990). The complements and immunoglobulins in different media of healthy pregnant women and in pregnant women with increased blood pressure. Clin Exp Obstet Gynecol.

[14] Hood JL, San RS, Wickline SA (2011). Exosomes released by melanoma cells prepare sentinel lymph nodes for tumor metastasis. Cancer Res.

[15] Du L, Yamamoto S, Burnette BL, Huang D, Gao K, Jamshidi N (2016). Transcriptome profiling reveals novel gene expression signatures and regulating transcription factors of TGFbeta-induced epithelial-to-mesenchymal transition. Cancer Med.

[16] Li LP, Lu CH, Chen ZP, Ge F, Wang T, Wang W (2011). Subcellular proteomics revealed the epithelial-mesenchymal transition phenotype in lung cancer. Proteomics.

[17] Brabletz T, Kalluri R, Nieto MA, Weinberg RA (2018). EMT in cancer. Nat Rev Cancer.

[18] Ansieau S, Bastid J, Doreau A, Morel AP, Bouchet BP, Thomas C (2008). Induction of EMT by twist proteins as a collateral effect of tumor-promoting inactivation of premature senescence. Cancer Cell.

[19] Ceausu AR, Ciolofan A, Cimpean AM, Magheti A, Mederle O, Raica M (2018). The mesenchymal-epithelial and epithelial-mesenchymal cellular plasticity of liver metastases with digestive origin. Anticancer Res.

[20] Jo M, Lester RD, Montel V, Eastman B, Takimoto S, Gonias SL (2009). Reversibility of epithelial-mesenchymal transition (EMT) induced in breast cancer cells by activation of urokinase receptor-dependent cell signaling. J Biol Chem.

[21] Kalluri R, Neilson EG (2003). Epithelial-mesenchymal transition and its implications for fibrosis. J Clin Invest.

[22] Zarnegar B, Mendez-Enriquez E, Westin A, Soderberg C, Dahlin JS, Gronvik KO (2017). Influenza infection in mice induces accumulation of lung mast cells through the recruitment and maturation of mast cell progenitors. Front Immunol.

[23] Kasai H, Allen JT, Mason RM, Kamimura T, Zhang Z (2005). TGF-beta1 induces human alveolar epithelial to mesenchymal cell transition (EMT). Respir Res.

[24] Yasukawa A, Hosoki K, Toda M, Miyake Y, Matsushima Y, Matsumoto T (2013). Eosinophils promote epithelial to mesenchymal transition of bronchial epithelial cells. PLoS One.

[25] Che D, Zhang S, Jing Z, Shang L, Jin S, Liu F (2017). Macrophages induce EMT to promote invasion of lung cancer cells through the IL-6-mediated COX-2/PGE2/beta-catenin signalling pathway. Mol Immunol.

[26] Kim KK, Kugler MC, Wolters PJ, Robillard L, Galvez MG, Brumwell AN (2006). Alveolar epithelial cell mesenchymal transition develops in vivo during pulmonary fibrosis and is regulated by the extracellular matrix. Proc Natl Acad Sci U S A.

[27] John G, Kohse K, Orasche J, Reda A, Schnelle-Kreis J, Zimmermann R (2014). The composition of cigarette smoke determines inflammatory cell recruitment to the lung in COPD mouse models. Clin Sci (Lond).

[28] McKnight CG, Jude JA, Zhu Z, Panettieri RA, Finkelman FD (2017). House dust mite-induced allergic airway disease is independent of IgE and FcepsilonRIalpha. Am J Respir Cell Mol Biol.

[29] Siddiqui S, Martin JG (2008). Structural aspects of airway remodeling in asthma. Curr Allergy Asthma Rep.

[30] Jiang Y, Wu Y, Hardie WJ, Zhou X (2017). Mast cell chymase affects the proliferation and metastasis of lung carcinoma cells in vitro. Oncol Lett.

[31] Allahverdian S, Harada N, Singhera GK, Knight DA, Dorscheid DR (2008). Secretion of IL-13 by airway epithelial cells enhances epithelial repair via HB-EGF. Am J Respir Cell Mol Biol.

[32] Luo D, Hu S, Tang C, Liu G (2018). Mesenchymal stem cells promote cell invasion and migration and autophagy-induced epithelial-mesenchymal transition in A549 lung adenocarcinoma cells. Cell Biochem Funct.

[33] Xiao H, Lasser C, Shelke GV, Wang J, Radinger M, Lunavat TR (2014). Mast cell exosomes promote lung adenocarcinoma cell proliferation - role of KIT-stem cell factor signaling. Cell Commun Signal.

[34] Yanan Y, Shelke GV, Jang SC, Lässer C, Wennmalm S, Hoffmann HJ (2017). Regulation of mesenchymal stem cell function by TGFβ-1 on mast cell extracellular vesicles — role of endosomal retention. bioRxiv.

[35] Sundstrom M, Vliagoftis H, Karlberg P, Butterfield JH, Nilsson K, Metcalfe DD (2003). Functional and phenotypic studies of two variants of a human mast cell line with a distinct set of mutations in the c-kit proto-oncogene. Immunology.

[36] Shelke GV, Lasser C, Gho YS, Lotvall J (2014). Importance of exosome depletion protocols to eliminate functional and RNA-containing extracellular vesicles from fetal bovine serum. J Extracell Vesicles.

[37] Bourboulia D, Stetler-Stevenson WG (2010). Matrix metalloproteinases (MMPs) and tissue inhibitors of metalloproteinases (TIMPs): positive and negative regulators in tumor cell adhesion. Semin Cancer Biol.

[38] Lanone S, Zheng T, Zhu Z, Liu W, Lee CG, Ma B (2002). Overlapping and enzyme-specific contributions of matrix metalloproteinases-9 and -12 in IL-13-induced inflammation and remodeling. J Clin Invest.

[39] Ayinde O, Wang Z, Griffin M (2017). Tissue transglutaminase induces epithelial-mesenchymal-transition and the acquisition of stem cell like characteristics in colorectal cancer cells. Oncotarget.

[40] Kim ES, Sohn YW, Moon A (2007). TGF-beta-induced transcriptional activation of MMP-2 is mediated by activating transcription factor (ATF)2 in human breast epithelial cells. Cancer Lett.

[41] Kroepil F, Fluegen G, Totikov Z, Baldus SE, Vay C, Schauer M (2012). Down-regulation of CDH1 is associated with expression of SNAI1 in colorectal adenomas. PLoS One.

[42] Dokic D, Dettman RW (2006). VCAM-1 inhibits TGFbeta stimulated epithelial-mesenchymal transformation by modulating rho activity and stabilizing intercellular adhesion in epicardial mesothelial cells. Dev Biol.

[43] Lin RY, Sullivan KM, Argenta PA, Meuli M, Lorenz HP, Adzick NS (1995). Exogenous transforming growth factor-beta amplifies its own expression and induces scar formation in a model of human fetal skin repair. Ann Surg.

[44] Heard BE, Nunn AJ, Kay AB (1989). Mast cells in human lungs. J Pathol.

[45] Cairns JA, Walls AF (1996). Mast cell tryptase is a mitogen for epithelial cells. Stimulation of IL-8 production and intercellular adhesion molecule-1 expression. J Immunol.

[46] de Boer WI, van Schadewijk A, Sont JK, Sharma HS, Stolk J, Hiemstra PS (1998). Transforming growth factor beta1 and recruitment of macrophages and mast cells in airways in chronic obstructive pulmonary disease. Am J Respir Crit Care Med.

[47] Kim DK, Cho YE, Komarow HD, Bandara G, Song BJ, Olivera A (2018). Mastocytosis-derived extracellular vesicles exhibit a mast cell signature, transfer KIT to stellate cells, and promote their activation. Proc Natl Acad Sci U S A.

[48] Lu J, Wu J, Tian J, Wang S (2018). Role of T cell-derived exosomes in immunoregulation. Immunol Res.

[49] Veerappan A, Thompson M, Savage AR, Silverman ML, Chan WS, Sung B (2016). Mast cells and exosomes in hyperoxia-induced neonatal lung disease. Am J Phys Lung Cell Mol Phys.

[50] Malina RM, Zavaleta AN (1976). Androgyny of physique in female track and field athletes. Ann Hum Biol.

[51] Coussens LM, Raymond WW, Bergers G, Laig-Webster M, Behrendtsen O, Werb Z (1999). Inflammatory mast cells up-regulate angiogenesis during squamous epithelial carcinogenesis. Genes Dev.

[52] Jung M, Lord MS, Cheng B, Lyons JG, Alkhouri H, Hughes JM (2013). Mast cells produce novel shorter forms of perlecan that contain functional endorepellin: a role in angiogenesis and wound healing. J Biol Chem.

[53] Shelke GV, Yin Y, Jang SC, Lasser C, Wennmalm S, Hoffmann HJ (2019). Endosomal signalling via exosome surface TGFbeta-1. J Extracell Vesicles.

[54] Lyon M, Rushton G, Gallagher JT (1997). The interaction of the transforming growth factor-betas with heparin/heparan sulfate is isoform-specific. J Biol Chem.

[55] Shute JK, Solic N, Shimizu J, McConnell W, Redington AE, Howarth PH (2004). Epithelial expression and release of FGF-2 from heparan sulphate binding sites in bronchial tissue in asthma. Thorax.

[56] Tessler S, Rockwell P, Hicklin D, Cohen T, Levi BZ, Witte L (1994). Heparin modulates the interaction of VEGF165 with soluble and cell associated flk-1 receptors. J Biol Chem.

[57] Shelke GV, Jang SC, Yanan Y, Lässer C, Lötvall J (2016). Human mast cells release extracellular vesicle-associated DNA. Matters.

[58] Maret D, Gruzglin E, Sadr MS, Siu V, Shan W, Koch AW (2010). Surface expression of precursor N-cadherin promotes tumor cell invasion. Neoplasia.

[59] Li Y, Li A, Junge J, Bronner M (2017). Planar cell polarity signaling coordinates oriented cell division and cell rearrangement in clonally expanding growth plate cartilage. Elife.

