# Supplementary material for: Extracellular vesicles from mast cells induce mesenchymal transition in airway epithelial cells
Source: Respir Res. 2020 May 1;21:101. doi: 10.1186/s12931-020-01346-8 (PMC7193353; doi:10.1186/s12931-020-01346-8)
Supplement: Supplementary file 1 — Additional file 1: Figure S1. Activation of SMAD in the bronchial epithelial cell line BEAS-2B after treatment with 30 μg/ml of EVs at 24 and 48 h. Table S1. Selected KEGG pathways related to proteins with differential abundance in epithelial A549 cells with or without EV treatment. Table S2. Selected cellular components related to proteins with differential abundance in epithelial A549 cells with or without EV treatment. The protein count indicates the number of proteins shown to engage with those cellular components. [file 12931_2020_1346_MOESM1_ESM.pdf]

# Supplementary Figure. 1

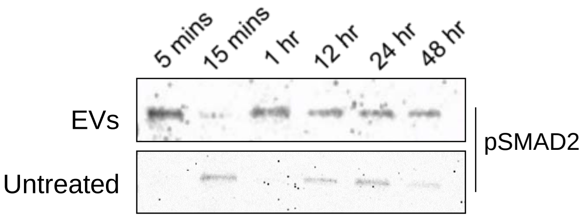

# Supplementary Table 1

| | Pathway ID | Pathway Description | Protein Count |
|---|---|---|---|
| 1 | 4151 | PI3K-Akt signaling pathway | 13 |
| 2 | 5200 | Pathways in cancer | 12 |
| 3 | 4066 | HIF-1 signaling pathway | 10 |
| 4 | 5206 | MicroRNAs in cancer | 9 |
| 5 | 4060 | Cytokine-cytokine receptor interaction | 9 |
| 6 | 4640 | Hematopoietic cell lineage | 8 |
| 7 | 4064 | NF-kappa B signaling pathway | 8 |
| 8 | 4630 | Jak-STAT signaling pathway | 8 |
| 9 | 4510 | Focal adhesion | 8 |
| 10 | 4668 | TNF signaling pathway | 7 |
| 11 | 4068 | FoxO signaling pathway | 7 |
| 12 | 4380 | Osteoclast differentiation | 7 |
| 13 | 4514 | Cell adhesion molecules (CAMs) | 7 |
| 14 | 5202 | Transcriptional misregulation in cancer | 7 |
| 15 | 4660 | T cell receptor signaling pathway | 6 |
| 16 | 4620 | Toll-like receptor signaling pathway | 6 |
| 17 | 5205 | Proteoglycans in cancer | 6 |
| 18 | 4014 | Ras signaling pathway | 6 |
| 19 | 4662 | B cell receptor signaling pathway | 5 |
| 20 | 5322 | Systemic lupus erythematosus | 5 |
| 21 | 4530 | Tight junction | 5 |
| 22 | 4015 | Rap1 signaling pathway | 5 |
| 23 | 4010 | MAPK signaling pathway | 5 |
| 24 | 4672 | Intestinal immune network for IgA production | 4 |
| 25 | 4621 | NOD-like receptor signaling pathway | 4 |
| 26 | 4623 | Cytosolic DNA-sensing pathway | 4 |
| 27 | 5212 | Pancreatic cancer | 4 |
| 28 | 4917 | Prolactin signaling pathway | 4 |
| 29 | 5222 | Small cell lung cancer | 4 |
| 30 | 4110 | Cell cycle | 4 |
| 31 | 4650 | Natural killer cell mediated cytotoxicity | 4 |
| 32 | 4145 | Phagosome | 4 |
| 33 | 4062 | Chemokine signaling pathway | 4 |
| 34 | 4144 | Endocytosis | 4 |
| 35 | 20 | Citrate cycle (TCA cycle) | 3 |
| 36 | 5330 | Allograft rejection | 3 |
| 37 | 4370 | VEGF signaling pathway | 3 |
| 38 | 4115 | p53 signaling pathway | 3 |
| 39 | 4612 | Antigen processing and presentation | 3 |
| 40 | 4622 | RIG-I-like receptor signaling pathway | 3 |
| 41 | 4914 | Progesterone-mediated oocyte maturation | 3 |
| 42 | 1200 | Carbon metabolism | 3 |
| 43 | 4725 | Cholinergic synapse | 3 |
| 44 | 3440 | Homologous recombination | 2 |
| 45 | 3410 | Base excision repair | 2 |
| 46 | 5340 | Primary immunodeficiency | 2 |
| 47 | 4960 | Aldosterone-regulated sodium reabsorption | 2 |
| 48 | 5219 | Bladder cancer | 2 |

# Supplementary Table 2

| | Pathway ID | Cellular Component | Protein Count |
|---|---|---|---|
| 1 | GO.0043227 | Membrane-bounded Organelle | 66 |
| 2 | GO.0044444 | Cytoplasmic Part | 47 |
| 3 | GO.0031988 | Membrane-bounded Vesicle | 38 |
| 4 | GO.0031982 | Vesicle | 37 |
| 5 | GO.0044421 | Extracellular Region Part | 36 |
| 6 | GO.0043233 | Organelle Lumen | 35 |
| 7 | GO.0005576 | Extracellular Region | 35 |
| 8 | GO.0070062 | Extracellular Exosome | 31 |
| 9 | GO.0005829 | Cytosol | 30 |
| 10 | GO.0005615 | Extracellular Space | 22 |
| 11 | GO.0009986 | Cell Surface | 17 |
| 12 | GO.0009897 | External Side Of Plasma Membrane | 15 |
| 13 | GO.0098552 | Side Of Membrane | 14 |
| 14 | GO.0016023 | Cytoplasmic Membrane-bounded Vesicle | 14 |
| 15 | GO.0030141 | Secretory Granule | 9 |
| 16 | GO.0031091 | Platelet Alpha Granule | 7 |
| 17 | GO.0031093 | Platelet Alpha Granule Lumen | 6 |
| 18 | GO.0045121 | Membrane Raft | 6 |
| 19 | GO.0005604 | Basement Membrane | 5 |
| 20 | GO.0005749 | Mitochondrial Respiratory Chain Complex II (Ubiquinone) | 3 |
| 21 | GO.0045273 | Respiratory Chain Complex II | 3 |
